# Platypnea‐Orthodeoxia Syndrome Post‐Pneumonectomy: A Case of Right‐To‐Left Shunting and Successful PFO Closure

**DOI:** 10.1002/ccr3.70715

**Published:** 2025-08-03

**Authors:** Cristian Castillo‐Rodriguez, Dina Soliman, Michel Juarez, Sherif Roman, John Abdelmalek, Zhaunn Sly, Ankush Lahoti, Victor Test

**Affiliations:** ^1^ Department of Internal Medicine Texas Tech University Health Sciences Center Lubbock Texas USA; ^2^ Division of Cardiology Texas Tech University Health Sciences Center Lubbock Texas USA; ^3^ Division of Pulmonary and Critical Care Texas Tech University Health Sciences Center Lubbock Texas USA

**Keywords:** atrial septal defect, orthodeoxia, patent foramen ovale, platypnea

## Abstract

Platypnea‐orthodeoxia syndrome is a rare disorder characterized by dyspnea (platypnea) and arterial desaturation (orthodeoxia) in the upright position, with symptom relief upon lying down. This syndrome is commonly associated with cardiac anomalies, particularly patent foramen ovale (PFO), where increased right atrial pressure facilitates right‐to‐left shunting, leading to hypoxemia. Other associated conditions include cirrhosis, pericardial effusion, and pneumonectomy. We present a 32‐year‐old female with a history of right pneumonectomy for invasive aspergillosis who presented with worsening dyspnea and a greater than 10% drop in SpO_2_ when seated compared to the supine position. Initial imaging demonstrated rightward displacement of thoracic structures, including the right atrium, but a transthoracic echocardiogram was negative for intracardiac shunt. Due to persistent hypoxemia, a transesophageal echocardiogram was performed, revealing a right‐to‐left shunt, an atrial septal aneurysm, and a prominent Eustachian valve. The patient underwent successful PFO closure, leading to the complete resolution of both dyspnea and hypoxemia. This case emphasizes the importance of comprehensive diagnostic evaluation in patients with POS, especially those with complex cardiopulmonary histories.

**Summary**

- Platypnea‐orthodeoxia syndrome (POS) is often misdiagnosed as positional oxygen saturation measurements are not routinely performed.
- It is crucial to consider POS in patients with altered anatomy, such as post‐pneumonectomy.
- Transesophageal echocardiography (TEE) is the gold standard for diagnosing intracardiac shunts, with shunt closure providing definitive treatment.

## 1 Introduction

Platypnea‐orthodeoxia syndrome (POS) is a rare condition marked by dyspnea and arterial desaturation when a patient is upright, with symptoms improving when lying down. This syndrome can arise from a right‐to‐left interatrial shunt, often through a patent foramen ovale (PFO), which can be aggravated by anatomical changes following pneumonectomy. After a pneumonectomy, particularly a right‐sided one, the shift in the mediastinum and changes in intrathoracic pressure can lead to the reopening or worsening of a PFO, contributing to POS. Several case reports have highlighted this phenomenon with remarkable improvement following surgical closure of the PFO [1, 2]. The diagnosis of POS typically involves pulse oximetry to confirm orthodeoxia and imaging studies, including transesophageal echocardiography (TEE) with a bubble study to visualize the shunt. Treatment usually involves closing the PFO, which can be done surgically or percutaneously, both of which are effective in resolving symptoms [3].

## 2 Case History and Physical Examination

A 32‐year‐old female with a history of right pneumonectomy due to right lower lobe cavitary lesions secondary to *Aspergillus fumigatus*. She presented 4 months after pneumonectomy due to worsening shortness of breath. She reported that her shortness of breath (SOB) had progressively worsened over the past 2 weeks and became unbearable in the last week. The dyspnea is exacerbated when transitioning from a lying to seated position or standing.

On physical examination, she appeared alert but in moderate respiratory distress. Respiratory assessment revealed increased work of breathing and diminished breath sounds on the right side, consistent with prior pneumonectomy. No wheezing or crackles were noted on the left lung. Cardiovascular examination was unremarkable, with normal heart sounds, no murmurs, no jugular venous distension (JVD), and no peripheral edema. Her oxygen saturation was 99% on high flow nasal cannula at a rate of 40 L and FiO_2_ of 60%. Tachycardia was noted; heart rate was 118 bpm. She was otherwise afebrile and normotensive. There was no positional SpO_2_ recorded at this point.

## 3 Differential Diagnosis, Investigations and Treatment

The differential diagnoses included post‐pneumonectomy syndrome due to mediastinal shift leading to airway compression, pulmonary hypertension secondary to single lung physiology, left lung compensatory failure or infection, pulmonary embolism, and cardiac‐related dyspnea such as heart failure or pericardial effusion.

**FIGURE 1:**
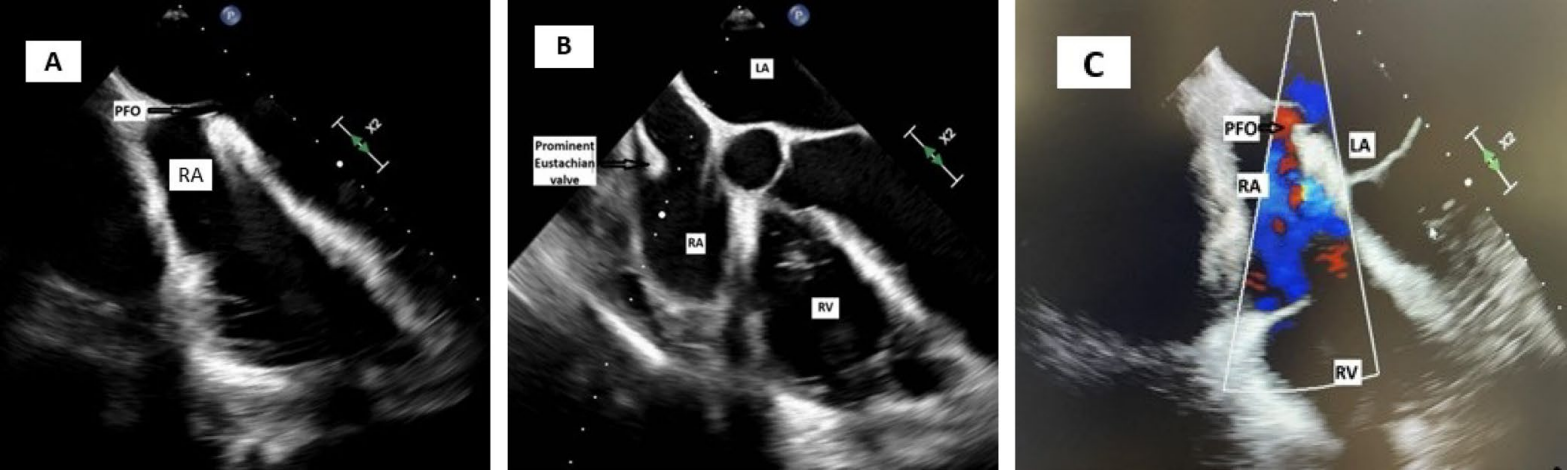
(A) A 2D‐TEE in sitting position, at the top of the imaging, an arrow shows the PFO. (B) A prominent Eustachian valve. (C) Using color Doppler there is clear evidence of the PFO.

**FIGURE 2:**
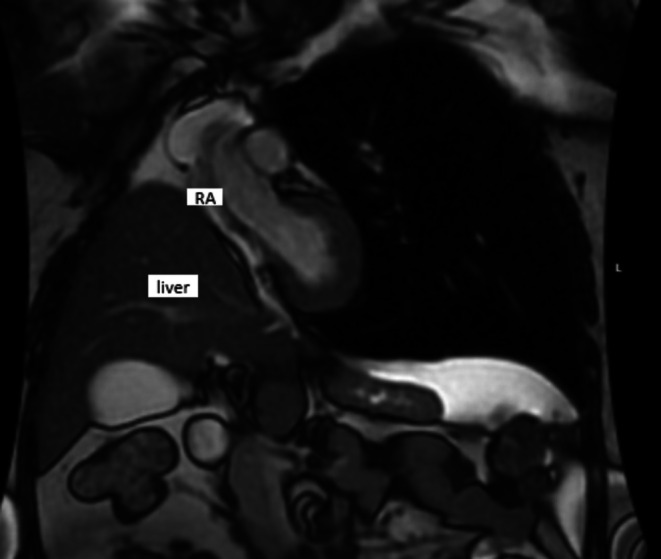
Cardiac MRI in the supine position showing remarkable right atrium collapse due to upward shift of the liver inside of the thoracic cavity.

A contrast‐enhanced chest CT showed no evidence of pulmonary embolism. The initial transthoracic echocardiogram (TTE) revealed paradoxical interventricular septal motion; the color Doppler examination and the agitated saline challenge test showed no evidence of an intracardiac shunt. Due to persistent hypoxemia, a bronchoscopy was performed for suspicion of postpneumonectomy syndrome, which did not show abnormalities. In addition, a V/Q scan was performed to assess vascular obstruction, which was normal. Findings from right heart catheterization ruled out the presence of pulmonary hypertension; interestingly, right heart hemodynamics were normal (Table 1). As the patient gradually weaned off oxygen during her hospitalization, we observed a positional difference in her oxygen saturation when transitioning to a seated or standing position. While seated, her oxygen saturation on room air was 80%, but it increased to 95% when supine, showing a drop of more than 10% in SpO_2_ upon sitting. This prompted further investigation for a potential cardiac shunt. A TEE was done with the patient sitting up in bed, and it was significant for right to left shunt due to PFO with an aneurysmal septum and a long tunnel (Figure 1A–C). Notably, a TEE was not performed during the pneumonectomy for comparison. A cardiac MRI showed a right atrial collapse (Figure 2).

**TABLE 1:** Right heart catheterization hemodynamic values.

Parameter	Value
Right internal jugular O_2_ saturation	69%
Right atrial pressure	2 mmHg
Right atrial O_2_ saturation	75%
Right ventricular pressure	25/0 mmHg
Right ventricular O_2_ saturation	72%
Pulmonary artery pressure	27/7 mmHg (mean 14 mmHg)
Pulmonary artery O_2_ saturation	71%
Pulmonary artery occlusion pressure	5 mmHg
Cardiac output (thermodilution)	5.26 L/min
Cardiac index	2.8 L/min/m^2^
Pulmonary vascular resistance	1.7 Woods units

**FIGURE 3:**
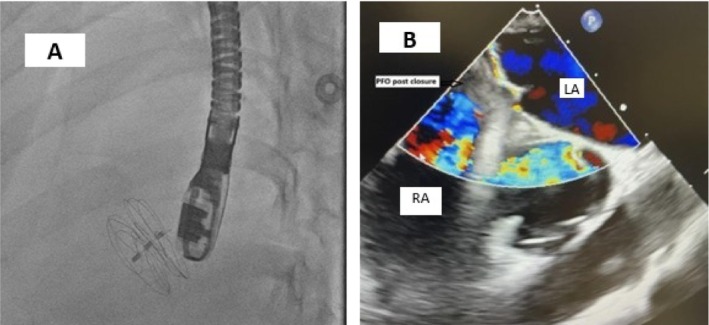
(A) Placement of the Gore septal occluder guided by TEE. (B) Color Doppler TEE in supine position, with a black arrow showing complete resolution of the PFO.

After a multidisciplinary discussion, the decision was made to perform percutaneous PFO closure. Due to the complex anatomy of the PFO with a long tunnel, as well as significant RA compression, initial plans for intracardiac echocardiography‐guided (ICE‐guided) PFO closure under sedation could not be performed as the interatrial septum could not be adequately visualized with the ICE catheter. Then, under general anesthesia, an intraoperative TEE was used with successful closure of the PFO with a GORE CARDIOFORM 30 mm Septal Occluder (W. L. Gore & Associates, Flagstaff, AZ, USA) (Figure 3A,B).

## 4 Results

The patient's symptoms fully resolved following the PFO closure. She was discharged without any signs of complications related to the procedure or dysfunction of the device.

## 5 Discussion

POS is a rare disorder characterized by dyspnea (platypnea) and arterial desaturation (orthodeoxia) when a patient is in the upright position and with improvement in the supine position [4]. Small physiological right‐to‐left shunts occur in the circulatory system without significant hemodynamic effects. In healthy individuals, bronchial arteries receive 2%–3% of cardiac output, with most of this returning to pulmonary veins. Additionally, thebesian veins drain a minimal amount (0.3%) into the left ventricle [5].

The primary pathophysiological process in POS involves the mixing of deoxygenated venous blood with arterial blood. In POS, deoxygenated blood bypasses the lungs and enters the systemic circulation, leading to hypoxemia. The severity of symptoms depends on the volume of blood shunted. A 20%–25% shunt fraction can lower arterial oxygen pressure below 70 mmHg, while severe hypoxemia (PaO2 < 40 mmHg) requires a 50% shunt fraction [6]. The right‐to‐left shunt seen in POS occurs fundamentally through two mechanisms, either intracardiac or intrapulmonary. Of these, the intracardiac mechanism is the most prevalent, accounting for over 80% of cases. This is predominantly attributed to a PFO, though less frequently, it may be caused by an atrial septal defect or an atrial septal aneurysm. While the occurrence of a right‐to‐left shunt may appear straightforward, with blood flowing from the right atrium to the left atrium, a more detailed analysis reveals a complex pathophysiological process. The development of intracardiac POS necessitates a combination of both structural and physiological alterations. This is exemplified by the high prevalence of PFO in the general population, estimated to be approximately 25%–30%, varying with age, juxtaposed with the relatively low incidence of POS syndrome [7, 8]. Conversely, the intrapulmonary mechanism is associated with vascular abnormalities, such as capillary lung dilation and ventilation/perfusion (V/Q) mismatch, which are commonly observed in conditions such as pulmonary arteriovenous malformations (PAVM), hepatopulmonary syndrome (HPS), and various pulmonary parenchymal disorders [9, 10].

The development of platypnea‐orthodeoxia syndrome (POS) in this patient can be explained by the following mechanism. The upward displacement of the right hemidiaphragm after pneumonectomy allowed the liver to shift into the right hemithorax, compressing the right atrium and impairing venous return, particularly during expiration. This relatively increased right atrial pressure and redirected blood flow through the PFO, exacerbating right‐to‐left shunting, especially when upright, as the interatrial septum is more stretched, and the gravitational increase in venous return facilitates more blood flow through the foramen ovale. This effect was further intensified by the prominent eustachian valve, which clearly redirected blood from the IVC toward the foramen ovale (Videos 1, 2). Other mechanisms described in the literature, such as increased right‐sided cardiac pressures due to a reduction in pulmonary vascular bed volume after pneumonectomy, could contribute in other cases. However, right heart catheterization in our patient confirmed that this was not the case, as the shunting was purely anatomical and position‐dependent [7].

**VIDEO 1:** TEE mid‐esophageal aortic valve short axis view showing collapse of right atrium seen on TEE during expiratory phase of respiration due to relaxation of the right hemidiaphragm and right atrial compression by the liver. Video content can be viewed at https://onlinelibrary.wiley.com/doi/10.1002/ccr3.70715.

**VIDEO 2:** TEE mid‐esophageal 4 chamber view showing prominent Eustachian valve redirecting blood flow toward foramen ovale seen in diastolic phase of the cardiac cycle Video content can be viewed at https://onlinelibrary.wiley.com/doi/10.1002/ccr3.70715.

POS is diagnosed clinically, starting with a detailed history and physical examination. A drop in PaO_2_ of more than 4 mmHg or in SaO_2_ of more than 5% upon standing, which improves when supine, confirms the diagnosis [11]. Since an intracardiac shunt is the most common mechanism, the initial assessment is performed using transthoracic echocardiography with contrast and agitated saline bubble study. The early appearance of bubbles in the left atrium (within three to six cardiac cycles) indicates an intracardiac shunt, whereas a delayed appearance of bubbles suggests an extracardiac shunt, which typically occurs in the pulmonary vascular network [9]. If TTE results are inconclusive or negative but clinical suspicion remains high, transesophageal echocardiography (TEE) with contrast is recommended. TEE provides a more detailed evaluation of the interatrial septum, adjacent structures, and suitability for device closure [7, 12].

Despite advancements in diagnostic tools, POS remains frequently misdiagnosed due to its rarity. For instance, among 546 patients who underwent pneumonectomy, only three developed POS requiring PFO closure [13]. In our patient, the diagnostic process was particularly challenging. Unlike previously reported cases, she was younger [3, 13], and her initial shunt evaluation using TTE was negative, with normal RAP and PAP values (Table 1). Moreover, while RA compression was evident on MRI, it was also apparent in other imaging modalities, though dependent on the imaging plane. For instance, (Video 1), the TEE AV SAX view, clearly illustrates significant RA compression, contributing to blood shunting and the subsequent platypnea–orthodeoxia syndrome. Furthermore, due to the altered anatomy, ICE‐guided PFO closure was not feasible, necessitating the use of TEE for enhanced visualization.

Treatment of POS is mainly by reversing the underlying pathophysiology. In patients with intracardiac shunts, percutaneous closure has proved to be effective and has a safe profile. Percutaneous PFO closure is the most common procedure done (88%), followed by atrial septal defect and perforated atrial septal aneurysm. Data support PFO closure for prevention of cryptogenic stroke. However, limited data are available about POS. According to Tereshchenko et al., percutaneous endovascular closure is a safe and effective treatment for POS associated with patent foramen ovale, alleviating orthodeoxia and related symptoms [14]. In a single‐center case series in Toronto, PFO closure by either PFO or non‐PFO closure devices resulted in complete resolution of symptoms and hypoxemia in all 52 included in the study [15].

The decision to intervene depends on symptom severity and potential complications. Careful patient selection and discussion of risks, such as atrial fibrillation, device‐related thrombus, cardiac tamponade, device embolization, late erosion, and infective endocarditis, are essential. Procedural expertise, close monitoring, and diligent follow‐up are crucial for optimal outcomes [16].

In conclusion, platypnea‐orthodeoxia syndrome is a relatively rare clinical syndrome characterized by dyspnea and hypoxia in an upright position, most commonly caused by intracardiac shunt. PFO closure achieved significant improvement in symptoms by preventing right‐to‐left shunting, which enhances respiratory function. This case also highlights the importance of advanced imaging techniques in patients with unexplained hypoxemia and positional dyspnea.

## Author Contributions

**Cristian Castillo‐Rodriguez:** data curation, formal analysis, writing – original draft. **Dina Soliman:** software, writing – review and editing. **Michel Juarez:** visualization, writing – review and editing. **Sherif Roman:** conceptualization, supervision, validation, visualization. **John Abdelmalek:** conceptualization, methodology, supervision. **Zhaunn Sly:** methodology, supervision, validation. **Ankush Lahoti:** methodology, supervision, validation. **Victor Test:** methodology, supervision, validation.

## Ethics Statement

The authors have nothing to report.

## Consent

The patient has provided written informed consent for the publication of this case report.

## Conflicts of Interest

The authors declare no conflicts of interest.

## Data Availability

The data supporting this case report are available within the manuscript. Due to patient confidentiality and ethical considerations, additional data, including medical records and imaging, are not publicly available. Any further details may be provided upon reasonable request, subject to institutional and ethical approvals.

## References

[1] S. Delalieux , K. De Greef , J. Hendriks , P. Lauwers , B. Suys , and P. Van Schil , “Orthodeoxia‐Platypnea Syndrome Presenting as Paradoxical Peripheral Embolism,” Annals of Thoracic Surgery 85, no. 5 (2008): 1798–1800, 10.1016/j.athoracsur.2007.08.011. 18442594

[2] J. T. Knapper , J. Schultz , G. Das , and L. S. Sperling , “Cardiac Platypnea‐Orthodeoxia Syndrome: An Often Unrecognized Malady,” Clinical Cardiology 37, no. 10 (2014): 645–649, 10.1002/clc.22301. 24912004 PMC6649356

[3] M. K. Mojadidi , R. Gevorgyan , N. Noureddin , and J. M. Tobis , “The Effect of Patent Foramen Ovale Closure in Patients With Platypnea‐Orthodeoxia Syndrome,” Catheterization and Cardiovascular Interventions 86, no. 4 (2015): 701–707. 26063336 10.1002/ccd.25953

[4] C. Blanche , S. Noble , M. Roffi , et al., “Platypnea‐Orthodeoxia Syndrome in the Elderly Treated by Percutaneous Patent Foramen Ovale Closure: A Case Series and Literature Review,” European Journal of Internal Medicine 24, no. 8 (2013): 813–817, 10.1016/j.ejim.2013.08.698. 24007641

[5] F. Saremi , H. Muresian , and D. Sánchez‐Quintana , “Coronary Veins: Comprehensive CT‐Anatomic Classification and Review of Variants and Clinical Implications,” Radiographics 32, no. 1 (2012): E1–E32. 22236907 10.1148/rg.321115014

[6] C. L. Shovlin , “Pulmonary Arteriovenous Malformations,” American Journal of Respiratory and Critical Care Medicine 190, no. 11 (2014): 1217–1228. 25420112 10.1164/rccm.201407-1254CI PMC4315816

[7] J. L. Salas‐Pacheco , “Mechanisms of Platypnea‐Orthodeoxia Syndrome,” Archivos de Cardiología de México (English Ed. Internet) 92, no. 2 (2022): 274–282. 10.24875/ACM.21000171 PMC9005182 34428199

[8] P. Lechat , J. L. Mas , G. Lascault , et al., “Prevalence of Patent Foramen Ovale in Patients With Stroke,” New England Journal of Medicine 318, no. 18 (1988): 1148–1152, 10.1056/nejm198805053181802. 3362165

[9] P. Rodrigues , P. Palma , and L. Sousa‐Pereira , “Platypnea‐Orthodeoxia Syndrome in Review: Defining a New Disease?,” Cardiology 123, no. 1 (2012): 15–23. 22948714 10.1159/000339872

[10] T. O. Cheng , “Platypnea‐Orthodeoxia Syndrome: Etiology, Differential Diagnosis, and Management,” Catheterization and Cardiovascular Interventions 47, no. 1 (1999): 64–66, 10.1002/(sici)1522-726x(199905)47:1<64::Aid-ccd15>3.0.Co;2-6. 10385164

[11] A. Agrawal , A. Palkar , and A. Talwar , “The Multiple Dimensions of Platypnea‐Orthodeoxia Syndrome: A Review,” Respiratory Medicine 129 (2017): 31–38. 28732833 10.1016/j.rmed.2017.05.016

[12] F. E. Silvestry , M. S. Cohen , L. B. Armsby , et al., “Guidelines for the Echocardiographic Assessment of Atrial Septal Defect and Patent Foramen Ovale: From the American Society of Echocardiography and Society for Cardiac Angiography and Interventions,” Journal of the American Society of Echocardiography 28, no. 8 (2015): 910–958, 10.1016/j.echo.2015.05.015. 26239900

[13] C. Aigner , G. Lang , S. Taghavi , et al., “Haemodynamic Complications After Pneumonectomy: Atrial Inflow Obstruction and Reopening of the Foramen Ovale,” European Journal of Cardio‐Thoracic Surgery 33, no. 2 (2008): 268–271. 18061472 10.1016/j.ejcts.2007.10.020

[14] A. S. Tereshchenko and E. V. Merkulov , “Modern Diagnostic Methods and Approaches to Treatment of Platypnea–Orthodeoxia Syndrome in Patients With Patent Foramen Ovale,” IP Pavlov Russian Medical Biological Herald 32, no. 4 (2024): 657–668.

[15] A. H. Shah , M. Osten , A. Leventhal , et al., “Percutaneous Intervention to Treat Platypnea–Orthodeoxia Syndrome: The Toronto Experience,” JACC. Cardiovascular Interventions 9, no. 18 (2016): 1928–1938. 27659570 10.1016/j.jcin.2016.07.003

[16] E. Horlick , C. J. Kavinsky , Z. Amin , et al., “SCAI Expert Consensus Statement on Operator and Institutional Requirements for PFO Closure for Secondary Prevention of Paradoxical Embolic Stroke: The American Academy of Neurology Affirms the Value of This Statement as an Educational Tool for Neurologists,” Catheterization and Cardiovascular Interventions 93, no. 5 (2019): 859–874, 10.1002/ccd.28111. 30896894

